# Technical approach to individualized respiratory-gated carbon-ion therapy for mobile organs

**DOI:** 10.1007/s12194-013-0208-3

**Published:** 2013-04-09

**Authors:** Mutsumi Tashiro, Takayoshi Ishii, Jun-ichi Koya, Ryosuke Okada, Yuji Kurosawa, Keisuke Arai, Satoshi Abe, Yoshiaki Ohashi, Hirofumi Shimada, Ken Yusa, Tatsuaki Kanai, Satoru Yamada, Hidemasa Kawamura, Takeshi Ebara, Tatsuya Ohno, Takashi Nakano

**Affiliations:** 1 Gunma University Heavy Ion Medical Center, 3-39-22 Showa-machi, Maebashi, Gunma 371-8511 Japan; 2 Present Address: Department of Radiation Oncology, Massachusetts General Hospital/Harvard Medical School, Boston, MA 02114 USA; 3 Present Address: Department of Radiation Oncology, Saitama Medical University International Medical Center, 1397-1 Yamane, Hidaka, Saitama 350-1298 Japan

**Keywords:** Carbon-ion therapy, Respiratory motion, Respiratory gating, Treatment planning, 4D CT, Errors

## Abstract

We propose a strategy of individualized image acquisitions and treatment planning for respiratory-gated carbon-ion therapy. We implemented it in clinical treatments for diseases of mobile organs such as lung cancers at the Gunma University Heavy Ion Medical Center in June 2010. Gated computed tomography (CT) scans were used for treatment planning, and four-dimensional (4D) CT scans were used to evaluate motion errors within the gating window to help define the internal margins (IMs) and planning target volume for each patient. The smearing technique or internal gross tumor volume (IGTV = GTV + IM), where the stopping power ratio was replaced with the tumor value, was used for range compensation of moving targets. Dose distributions were obtained using the gated CT images for the treatment plans. The influence of respiratory motion on the dose distribution was verified with the planned beam settings using 4D CT images at some phases within the gating window before the adoption of the plan. A total of 14 lung cancer patients were treated in the first year. The planned margins with the proposed method were verified with clinical X-ray set-up images by deriving setup and internal motion errors. The planned margins were considered to be reasonable compared with the errors, except for large errors observed in some cases.

## Introduction

Various techniques have been adopted for dealing with respiratory motion in radiation therapy [1–6]. The gating technique has been widely used for radiation and particle therapy. This technique appears to cause a low burden to patients, relatively simple system implementation, and reasonable expected efficacy. In particle therapy, the high-dose region is targeted not only by adjusting patient positioning in the lateral directions, but also by adjusting the spread-out Bragg peak (SOBP) width and the beam range in the beam-depth direction. Therefore, motion on the beam path and the resulting changes in the radiological path length to the target may cause considerable failure in dose coverage, with hot and cold spots occurring in the target and surrounding normal tissues.

In gated radiotherapy, the amount of respiratory motion differs for each patient even during the gating window. Accounting for the target position and the amount of motion during the gating window with appropriate treatment plans is required for better dose delivery. Medical images used for treatment planning and patient positioning should be obtained with the appropriate timing, where the delay between the gating system and the imaging has to be taken into account.

Generally, treatment planning adds margins to a clinical target volume (CTV) to compensate for uncertainties of the target position with respect to the irradiation field. Some formulations have quantified the planning target volume (PTV) margins [7–9]. In these cases, the standard deviations of systematic and random errors have to be known in a group of similar patients who were treated previously. However, such statistical patient data are limited and difficult to quantify for a new facility. Furthermore, the amount of respiratory motion and the tumor locations may vary significantly among individual patients. Therefore, the use of a specific amount of margin may cause larger or smaller PTVs than necessary. It is impossible to predict the precise internal margins for individual patients that undergo different motion during treatment. However, it is expected that such margins may be quantified with reasonable accuracy by measuring the motion directly from medical images acquired for individual patients.

We have developed a methodology to incorporate image acquisition into treatment planning for respiratory-gated carbon-ion therapy. This paper describes a sequence of carbon-ion treatment planning for respiratory-gated therapy to improve the dose coverage to a target and the dose suppression in surrounding normal tissues through appropriate margin setting and range compensator design. Some papers have reported on the delays of gating systems [10–12] and treatment planning strategies of particle beam therapy for mobile tumors [13, 14]. However, for respiratory gating, papers compiling the practical processes such as proposing the method for deriving appropriate timings of image acquisitions including delay times of the gating systems, subsequent margin setting and range compensator design in treatment planning, its clinical application, and verification of the planned margins with clinical experience have not been reported so far, especially for ion beam therapy.

Technically, intrinsic problems still remain, such as reproducibility of respiratory motion in gating and correlation between the motion and the monitored respiratory waveform, even though the intent is to use stable patient respiration. Currently, we must postulate such reproducibility and correlation to some extent to undertake the treatment. Although our approach is based on this hypothesis, it is practical for routine use with the present system and is expected to have reasonable accuracy.

Images necessary to take into account the motion during gating are acquired for each patient and are reflected in the treatment planning. Because gated CT and 4D CT images, used for treatment planning, are acquired using a monitored respiratory waveform, and the carbon beam irradiation is carried out also using respiratory waveforms, errors based on the correlation between respiratory waveform and organ motion are reduced. Inter-fractional setup errors are derived from the accuracy of the positioning system. Inter-fractional internal errors are suppressed as much as possible by keeping the patient’s anatomical condition stable with immobilization devices and pretreatment procedures since these are difficult to quantify for each patient, as well as intra-fractional setup errors. Intra-fractional internal errors, which may differ largely among patients, may be mostly taken into account for margin definitions by measuring the amount of motion for each patient.

We started carbon-ion therapy at the Gunma University Heavy Ion Medical Center (GHMC) in March 2010. Respiratory-gated therapy for mobile organs, such as lung and liver, started in June 2010. In this paper, we present our system and strategy for the treatment of mobile organs, especially for lung cancer treatment, and we present validation of the treatment methodology using daily positioning X-ray images for lung cancer patients in the first year.

## Materials and methods

### System

**Fig. 1:**
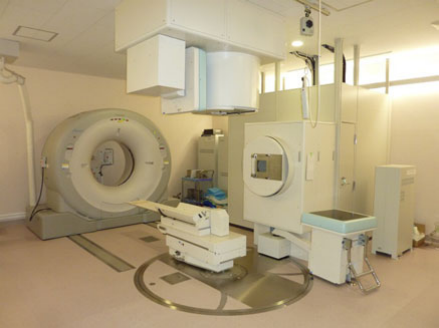
Photograph of CT simulation room

The carbon-ion therapy facility equipped with a broad-beam irradiation system and the typical treatment flow at GHMC are described elsewhere [15, 16]. Here, we describe the respiratory-gated therapy system. An X-ray CT (Toshiba Aquilion LB, self-propelled) for treatment planning and a couch having the same specification as that in the carbon beam irradiation rooms are located in the CT simulation room. There is also an X-ray TV (XTV) system, which uses Shimadzu X-ray tubes and flat-panel detectors (FPD) (frontal and lateral directions) with the same geometry as those in the irradiation rooms. The couch can be moved between CT scan and XTV modes by rotating the turntable about its center axis and the couch about the axis of the couch post. The X-ray tubes are equipped with mock irradiation ports, and geometric interference among the patient, the couch, and the irradiation nozzles can be checked to determine the nozzle positions for the carbon beams. A photograph of the CT simulation room is shown in Fig. 1. The respiratory gating system (Anzai Medical AZ-733 V with laser respiration sensor) is equipped with CT, XTV scanners for gated and cine image acquisitions, and a carbon beam irradiation system (Mitsubishi Electric, Tokyo, Japan).

To obtain images at the desired timing for gated irradiation, we measured the delay times in the gating system and between the gating system and the modalities, such as CT, XTV, and the carbon-ion irradiation system. We monitored the time differences of the signals on an oscilloscope between a waveform just below the laser sensor (response time of ~3.4 ms), corresponding to the motion of a respiratory motion phantom (QUASAR, Modus Medical Devices Inc., London, Canada), and an output respiratory waveform, and between the output waveform and a gated signal. The delay time for the gated images was derived from the shifted image position of the 1 mm steel ball between an image of the static phantom and an image gated at the static phantom position at constant speed. The center positions of the steel ball in the images were pointed to measure the shifts; therefore, the delay times of the gated images expressed the average times over the motion during CT scanning and X-ray exposure, respectively. Standard conditions for lung or liver cancer patients were used for CT and XTV image acquisitions. The timing of a gated carbon beam was confirmed by monitoring the gated signal and the dose monitor (secondary emission monitor, SEM) output signal of the carbon-ion beam (response time of <1 ms). Using the obtained delay times, the gating levels for image acquisitions were determined for each patient as described below.

### Image acquisition

For treatment planning, the gated CT scan was performed around the end of expiration because that phase was expected to be the most stable and reproducible during the respiratory cycle. For a patient immobilized on the couch, the respiratory waveform was monitored to ensure stability and was recorded. The gating level was determined to be the amplitude of the waveform corresponding to the time minus the delay time of the gated CT scan from the time at the end expiration. The acquisition parameters of the gated CT scan were 550 mm field of view (FOV) for trunk, 2 mm slice thickness, and 0.5 s/rotation scan speed.

**Fig. 2:**
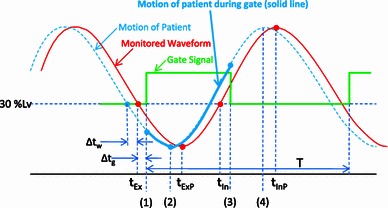
Relationship among respiratory motion of patient, monitored waveform, and gate signal. Reconstructed 4D CT phases are shown as* numbers*

A 4D CT scan was performed just after the gated CT scan to quantify the amount of motion and confirm the dose distributions during the gating window. The 4D CT projection data were acquired with a helical scan mode, as the CT gantry shifted slowly (typical helical pitch of 1.4–2 for 1 mm × 16 slices), and 4D CT images at the desired phases were reconstructed. The 4D CT images might include the motion artifacts in the time resolution of 0.3 s (time for half reconstruction) around the phases. The respiratory waveform and the gated signal were transmitted to the CT system. The respiration phase was defined as 0 %Ph when the gating signal was triggered and was expressed in percent of the interval to the next gate. Basically, the adopted gating level was 30 %Lv of the wave height, assuming a gated carbon-ion therapy situation. To distinguish the unit from phase (%Ph), here %Lv is used to express the amplitude level of a respiratory waveform, where 0 %Lv indicates the ideal exhalation peak and 100 %Lv indicates the ideal inhalation peak. For 4D CT images, reconstruction phases corresponding to exhalation 30 %Lv when the gating signal was triggered (gate-in), exhalation peak, inhalation 30 %Lv when the gating signal turned off (gate-out), and inhalation peak in a patient’s motion were derived as follows: As shown in Fig. 2, patient motion preceded the monitored respiratory waveform and the gated signal because of the delay time of the gating system. Therefore, time values *t*_Ex_, *t*_ExP_, *t*_In_, *t*_InP_, and *T* corresponding to 30 %Lv at exhalation, exhalation peak, 30 %Lv at inhalation, inhalation peak, and wave cycle, respectively, were read out from the typical and stable part of the respiratory waveform recorded simultaneously with the 4D CT scan. Reconstruction phases were then derived with the following formulae:

1. Exhalation 30 %Lv (gate-in): 0(%Ph),
2. Exhalation peak: $$ \frac{{t_{\text{ExP}} - t_{\text{Ex}} - (\Updelta t_{\text{w}} + \Updelta t_{\text{g}} )}}{T} \times 100\,(\% {\text{Ph),}} $$
3. Inhalation 30 %Lv (gate-out): $\frac{{t_{\text{In}} - t_{\text{Ex}} }}{T} \times 100\,(\% {\text{Ph),}}$ and
4. Inhalation peak: $$ \frac{{t_{\text{InP}} - t_{\text{Ex}} - (\Updelta t_{\text{w}} + \Updelta t_{\text{g}} )}}{T} \times 100\,(\% {\text{Ph),}} $$

where Δ*t*_w_ was the delay time between phantom motion and the respiratory wave (0.10 s) and Δ*t*_g_ was the delay time between the respiratory wave and the gating signal (0.11 s). The gating level was reduced, e.g., to 15 %Lv, when the amount of motion during the gating window was estimated to be large during the treatment planning stage. Corresponding 4D CT images were reconstructed.

### Treatment planning

Treatment planning includes the contouring of target volumes, the setting of margins and beam parameters, the designing of range compensators (RCs, bolus), and dose calculations. At our facility, XiO-N is used for treatment planning, which is XiO (Elekta)-based software incorporating a dose engine for ion beam radiotherapy (K2dose) [17–21] developed by the National Institute of Radiological Sciences (NIRS), Japan, with interfaces from Mitsubishi Electric.

#### Evaluation of motion and margins

For lung cancer, a CTV was contoured by adding, e.g., 8 mm margins to all directions from a gross tumor volume (GTV) and was limited within the lung.

The tumor motion in six directions was evaluated from 4D CT images relative to a gated CT image using Focal4D software (Elekta, Stockholm, Sweden). This was done by following the displacement of an anatomical feature such as the tumor edge or metal markers (gold sphere with 1.5 mm diameter) placed into the bronchus in advance. For simplicity, the motion during the gating window was assumed to be linear.

- Internal margin (IM)

Slightly larger IMs were set for a measured amount of motion to account for errors in the reproducibility of respiration and the correlation between the respiratory wave and the actual motion. Therefore, the IM was set to −10–40 %Lv motion by adding 10 %Lv when the gated motion was within 0–30 %Lv of all respiratory motion. Specifically, the IM was set by adding 1/3 of the motion during the gating window in each direction (superior–inferior (SI), left–right (LR), and anterior–posterior (AP)).

- Setup margin (SM)

The SM was determined as approximately 3 mm in all directions by taking the square root of the sum of the squares of the accuracies of the system shown in Table 1. The patient positioning error (so-called setup error) was supposed to be 2 mm including image-intrinsic errors, as shown in Table 1.

**Table 1:** Accuracies of the system used to determine setup margins for perpendicular-to-beam (a) and beam-axis (b) directions

(a) Perpendicular to beam direction (lateral)	
Displacement of beam axis	≦±0.5 mm
Position of RC	≦±0.5 mm
Position of patient collimator	≦±0.5 mm
Position of MLC	≦±0.5 mm
Displacement of ICs for horizontal and vertical beam courses	≦±0.5 mm
X-ray beam axis from IC	≦±0.5 mm
FPD axis from IC	≦±0.5 mm
Positioning system (image registration) (Δ*P*)	≦±2 mm
Machining accuracy of RC	≦±0.3 mm
Machining accuracy of patient collimator	≦±0.3 mm
(b) Beam axis direction	
CT conversion (2 %) for 15 cm (if 10 cm, ± 2 mm)	≦±3 mm
Range	≦±1 mm
Machining accuracy of RC	≦±0.3 mm
Uniformity of RC material (1 %) for 5 cm	≦±0.5 mm

- Total margin (TM)

The TM was determined from a combination of the IM and SM in each direction. Since the summation of IM and SM became large, and the possibility of such a maximum error was expected to be low (see Sect. 4), the TM was taken as the square root of the sum of their squares, rounded down to the nearest mm,

1 $$ {\text{TM = }}\sqrt {{\text{IM}}^{2} + {\text{SM}}^{2} } . $$

This value was used to define the PTV from the CTV using the auto-margin function of XiO-N.

We used the PTV margins for the lateral directions of the beam to determine the shape of the multi-leaf collimator (MLC) and the broadened beam size (the beam-wobbling radius and the scatterer thickness). The margins for each beam direction should also be given as the water-equivalent length because they originate in the accuracy of the beam penetration range. However, because a PTV definition for each beam direction made the planning procedure too cumbersome and complicated for routine work, the PTV was simply defined geometrically for all directions, independent of the beam directions. Furthermore, a proximal margin (PM) and/or a distal margin (DM) was added when the geometric margins for the beam directions were insufficient. Here, the PMs were added upstream from the target and the DMs were added downstream from the target for the beam direction as water-equivalent path length and affected the adoptions of the SOBP size, beam energy, and thickness of range shifters and design of RCs (described below). For example, in the case of the lung, a margin of 3 mm was added to the CTV in the lung field when the TM of the distal side was 3 mm. As the stopping power ratio of lung tissue to water was about 1/3, corresponding to a 1 mm margin in water-equivalent length, the residual 2 mm was set as DM. Such PM and DM values were determined for each case, depending on the anatomic structure around the target.

The MLC was composed of 40 pairs of iron leaves with 3.75 mm thickness and a maximum aperture size of 150 × 150 mm^2^. The position of the MLC, which was determined at treatment planning, could be adjusted in the range 250–670 mm from the iso-center (IC). The leaf margin was normally adjusted to 5–6 mm on the IC plane to cover the PTV with 95 % of a prescribed dose.

#### Design of range compensators

Range compensators (RCs) are normally used for broad-beam techniques in particle beam therapy [22]. The RC was made of polyethylene and fabricated to adjust the beam range across the target to conform the distal edge of the SOBP to the distal geometry of the target according to the patient anatomy. The grid size of the RCs was 3 mm. The thickness of the RC was calculated by subtracting the water-equivalent length from the patient surface to the distal part of the target from the range of the adopted beam source for each point in the beam’s-eye view. Furthermore, ‘smearing’ was applied to cover the range to the target for lateral positional errors [13, 23]. For the smearing procedure in XiO-N, rays from a virtual source point (center of the wobbler magnets) to corresponding points on the distal edge of a target in the lateral plane to the beam direction were considered. For each ray, the water-equivalent length from the patient surface to the proximal position of the target and that from the patient surface to the distal position of the target were calculated, and a target entrance depth distribution and a target exit depth distribution were derived as bitmap data. The values of the bitmap data were replaced to the minimum and maximum values within the smearing distance in the target entrance and exit depth distributions, respectively. The maximum value of the difference between the renewed target entrance and exit depth distributions for each ray was adopted as the required SOBP width, and the shape of the RC was derived from the renewed target exit depth distribution. The smearing distance was basically set to be the SM.

Even for structures with large density differences moving perpendicular to the beam, such as lung cancer, the high-dose region should be covered in the target in motion. Thus, we adopted two ways to design RCs for motion management, which were practical for clinical routine when we used our commercially available treatment planning system, XiO-N:

- Smearing method

The change of the range accompanied by motion was compensated for by setting smearing to be the TM perpendicular to the beam. Because the smearing value was not accepted for each direction in the XiO-N, the maximum value of the TMs perpendicular to the beam was adopted for the smearing distance. The change of the water-equivalent depth was compensated for by setting the PM and the DM to be the TM of the beam direction.

- IGTV method

The volume that a GTV was supposed to move during gated irradiation was defined as the internal gross tumor volume (IGTV) [14]. In practice, the IGTV was calculated by adding IM to the GTV for each direction. The RC was designed by replacing the stopping power ratio of the IGTV with an average value of the tumor, i.e., higher than the surrounding lung tissue. Here, since the motion was compensated by IGTV, the smearing values, PM and DM, were set equal to SM. After designing the RCs, the stopping power ratio was returned to the original value, and the dose distributions were recalculated. This was because the dose distributions should be confirmed with the beam penetration around the original GTV using the RCs designed for the IGTV.

Using RCs designed by these two methods, the dose distributions were calculated in XiO-N. Either method was adopted for a treatment plan case by case depending on the dose distributions and/or the amount of IM, as described in the Sect. 3. As our planning policy, when the range compensation was found to be insufficient in the dose distribution for each beam, the PM and/or the DM were adjusted to cover the CTV not only for gated CT images, but also for 4D CT images at phases in the gating window, as described below.

#### Calculation of dose distribution

Since the direction of the carbon-ion beam port in our facility was fixed to be either horizontal or vertical, dose concentration to a target was accomplished by rolling the patient on the couch, in other words, rotating along the SI axis, and thus increasing the beam directions for lung cancer patients. Typically, lung cancer treatments were performed with a total dose of 52.8 or 60 Gy (RBE), 4 fractions, 2 ports in a fraction, and 2 patient positions (±20° rolling with supine or prone position depending on the location of the tumor) [24]. CT scan and subsequent treatment planning were done for each patient position because the anatomic location of organs might change depending on the patient position. To confirm a total dose distribution between different patient positions, dose distributions of all beam ports were calculated in one position with virtual beams at another position. Using the planned beams on the gated CT images, dose distributions were calculated for 4D CT images at phases of gate-in, exhalation peak, and gate-out to confirm plan parameters including margins and RCs and the dose coverage to the CTV. When the amount of motion was small and gating was not estimated to be necessary, i.e., the IM for all respiratory motion was ~3 mm or less, the dose distribution was confirmed at a phase of the inhalation peak, and gating was not decided to be applied. The conversion table of CT value to stopping power ratio for gated CT images was confirmed in advance to be used for dose distribution calculation using 4D CT images.

### Patient positioning at irradiation

The XTV system for the frontal and lateral directions was used for patient positioning. The X-ray tube was 1595 mm and the FPD was 545 mm from the iso-center. The size of the FPD was 17 × 17 inch^2^ with 2880 × 2880 pixels and 12-bit density resolution. The image data 12 × 12 inch^2^ of the central area was sent to a positioning system and scaled down to 512 × 512 pixels (0.45 mm/pixel at iso-center plane) and 8-bit density resolution.

Patient positioning before irradiation was carried out using frontal and lateral XTV systems to adjust the patient couch. The gating level of a gated X-ray shot was determined by accounting for the delay time of the system, as with the gated CT scan, and was intended to be taken at the exhalation peak for each patient. The displacements were calculated for bony structures and metal markers with positioning software to confirm the matching of the set-up images to the reference images. For each direction, 10 feature points at maximum were manually extracted from bony structures such as the centrum, costa, scapula, and clavicle, or metal markers (3 points at maximum) for a set-up image and corresponding reference image. To validate the planned margins for gated lung cancer treatment, we evaluated setup errors for bony structure and internal motion errors for metal markers from the positioning error data. The analysis was conducted for a total of 14 lung cancer patients (4 images for each direction per patient) from the beginning of the gated therapy in June 2010 to July 2011.

## Results

The delay times in the gating system and between the gating system and the modalities, such as CT, XTV, and carbon-ion irradiation system are summarized in Table 2. These delay times were not considered to be negligible compared to general respiratory cycles and motion, except for that between the gating signal and the carbon beam irradiation.

**Table 2:** Delay time (seconds) between the respiratory gating system and other modalities

Phantom-Resp. wave output: Δ*t*_w_	0.10 s
Resp. wave out-gating signal: Δ*t*_g_	0.11 s
Phantom-gated CT image	0.62 s
Phantom-gated X-ray image	0.35 s
Gating signal-carbon beam irradiation	~0.001 s

**Fig. 3:**
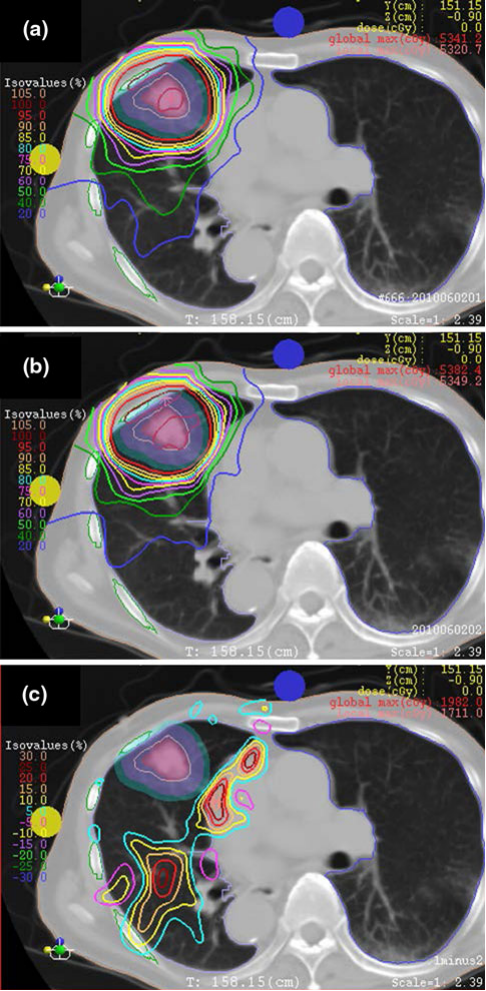
Examples of dose distributions using RCs designed by means of smearing (**a**) and IGTV (**b**) methods, and difference of the dose distributions, smearing minus IGTV methods (**c**)

Using RCs designed by the two methods, the dose distributions were calculated in XiO-N. An example of the dose distributions using the two methods and the difference between them is shown in Fig. 3. The dose homogeneity in a target was usually similar with both methods. Both plans were acceptable for clinical use. However, when the IM was large, the smearing was applied in all directions perpendicular to the beam, and the distal part of the target was influenced by the large smearing value. Therefore, the dose around the target could be suppressed more with the IGTV method. In this case, we employed the IGTV method. When the IM was small, the smearing value became similar to the SM. Therefore, the dose distributions were similar for both methods. In this case, we used the smearing method because of the simple planning procedure.

**Fig. 4:**
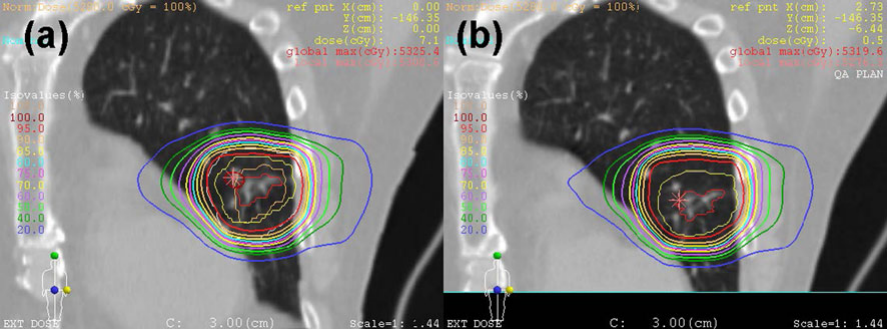
Examples of dose distributions for treatment planning (**a**) and 4D CT confirmation at gate-out (**b**). *Red*, *yellow*, *orange*, and *white* targets indicate GTV, CTV, IGTV, and PTV, respectively. The IGTV and PTV are contoured only on the gated CT images (**a**)

An example of dose distributions for treatment planning and 4D CT confirmation at a phase of gate-out is shown in Fig. 4. For all patients, the CTVs were almost covered with more than 95 % of the prescribed dose at phases in the gating window without adjustment of plan parameters such as PM and DM from the initially estimated values. The range compensation and dose coverage could be confirmed at treatment planning.

**Fig. 5:**
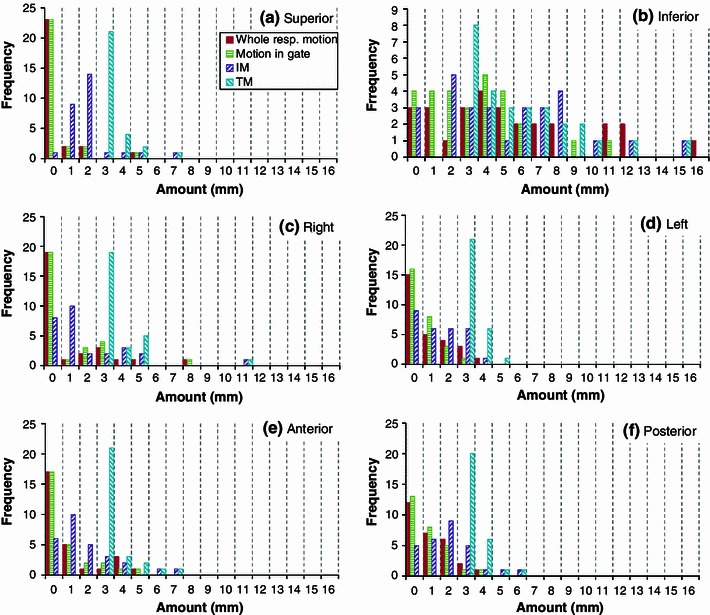
*Histograms* of the amounts of motion in whole respiration and gating, estimated IMs, and TMs for all patients and positions

Amounts of motion in whole respiration and gating, estimated IM, and TM for all patients and positions are shown in Fig. 5 as histograms for all directions. For 5 of the 14 patients, the gating was considered unnecessary because of the small amount of motion. In contrast, the gating level was decreased to 15 %Lv in one case because the amount of motion was large and was considered to influence the dose distribution in the surrounding normal tissues. The inferior motion had a large variation among patients. The maximum value of the inferior motion was 16 mm for whole respiration, and the motion during the gate was distributed mostly in the region 0–6 mm across a range 0–11 mm. The TMs of the inferior direction had a wide range of 3–15 mm including SM. The motion of the other directions peaked at 0 mm and was mostly distributed within a few mm. IMs of the superior direction were a little larger than those of the other directions since 1/3 of the superior-inferior motion was added to the measured superior motion for IM. TM, i.e., the PTV margin, was 3 mm or greater and mostly within 5 mm except in the inferior direction.

**Fig. 6:**
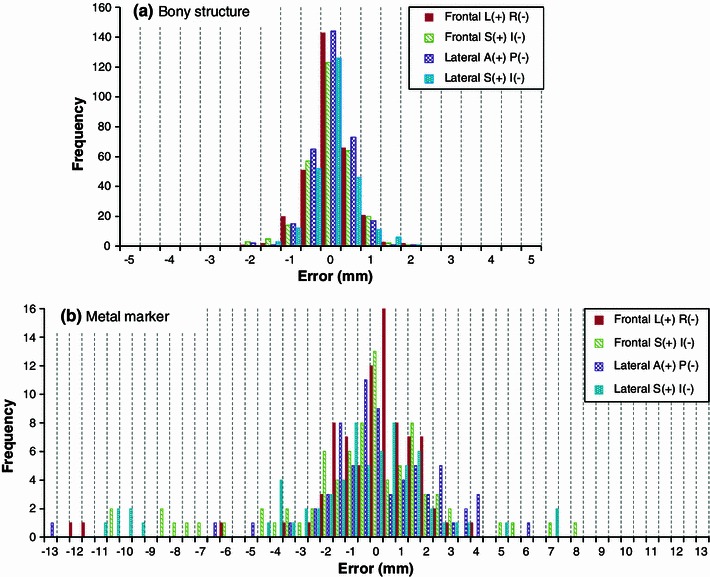
Error distributions of feature points of bony structures (**a**) and metal markers (**b**) on daily positioning X-ray images with respect to reference images

Error distributions of the feature points of bony structures and metal markers on daily positioning X-ray images with respect to reference images are shown in Fig. 6 for all directions [left (L), right (R), superior (S), inferior (I), anterior (A), and posterior (P) directions]. The statistics of the distributions are summarized in Table 3. The error values derived from the images correspond to those on the iso-center plane. The errors of bony structures correspond to setup errors in positioning. Errors of metal markers correspond to internal motion errors including positioning setup errors, which do not necessarily coincide with tumor motion because the metal markers are not placed in the tumor. However, because the metal marker errors indicate those of the tumor-surrounding volume including the target, target motion errors can be expected from the metal marker errors to some extent. It is important to comprehend errors of bony structures and tumor-surrounding volume including the target because not only the motion of the target but the change of anatomical structures along the beam path may influence the dose distribution.

**Table 3:** Errors of bony structures and metal markers at patient positioning

	Setup error of bony structure			Marker error		
	L(+) R(−)	S(+) I(−)	A(+) P(−)	L(+) R(−)	S(+) I(−)	A(+) P(−)
Number of points	309	546	319	82	151	69
Average	0.0	0.0	0.0	−0.1	−0.8	0.0
SD (*σ*) (mm)	0.5	0.5	0.5	2.4	3.5	2.6
Minimum (mm)	−1.9	−1.8	−1.9	−12.2	−11.2	−13.0
Maximum (mm)	1.9	2.1	2.1	3.9	8.1	5.8
SD fitted by L.S. (mm)				1.4	1.7	1.8

Data of superior–inferior (SI) direction are estimated using frontal and lateral images. The absolute values of the minimums indicate the maximum values of negative directions. The error values are those corresponding to the iso-center plane. The values in the bottom row indicate SDs of least-square (LS) fit of the marker error distribution (Fig. 6b) to a normal distribution (see text)

Setup errors of bony structures had a standard deviation (SD) of 0.5 mm in all directions. Marker errors had SDs of 2.4, 3.5, and 2.6 mm with averages of −0.1, −0.8, and 0.0 mm for the LR, SI, and AP directions, respectively, although the statistical precision was seen to be insufficient. The error distributions of many markers peaked around 0, although some markers were largely displaced. When a large displacement was found in lung anatomy positioning, two to three markers may have shifted similarly, leading to a large displacement on the histogram. The internal motion errors were normally about a few mm; therefore, they might be almost covered by smearing and/or IGTV procedures except for large errors observed in some cases.

## Discussion

### TM determination from SM and IM

The PTV margin, i.e., TM, is defined as the margin added to the CTV including SM and IM. Their simple summation often makes the PTV large, which safely covers positional errors [25]. However, clinical practice requires that the dose to normal tissue be suppressed using as small margins as possible. Therefore, we defined the TM in terms of Eq. (1) to determine PTV from CTV. However, the IM here was derived from the actual amount of motion from a single set of 4D CT images for each patient position, and we were not sure that it was directly correlated with the SD of the internal motion error. This TM was determined empirically; therefore, the adequacy of the TM should be validated by comparing with error data. The validity of our TM definition is discussed below.

**Fig. 7:**
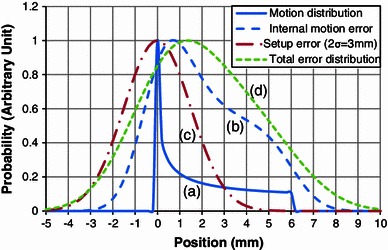
Error probability distributions of respiratory motion (assuming 6 mm in gating), internal motion error, setup error (2*σ* = 3 mm), and total error

Here, we consider the error probability distribution. In periodic motion, the probability that a point in a lung exists at a position between *x* and *x* + d*x* is proportional to the time interval d*t* corresponding to d*x*. Assuming respiratory motion to be sinusoidal, when gating is done at 30 %Lv at the exhalation side of the whole respiration and the amount of motion within the gate is, e.g., 6 mm, the probability distribution can be expressed as in Fig. 7a. Here, we see that the largest probability was at the exhalation peak. To determine IM, 1/3 of the gated motion was added to both directions to account for errors in the reproducibility of respiration and the correlation between the respiratory waveform and the actual motion (the IM of the positive direction is 8 mm). This was then incorporated as the convolution of the normal distribution with 2*σ* = 2 mm into the motion probability distribution, as shown in Fig. 7b. The setup error can be regarded as a random error having a normal probability distribution with 2*σ* = 3 mm in our system, as shown in Fig. 7c (SM = 3 mm). The probability distribution of the total error was considered to be the convolution of the internal motion and setup error distributions, as illustrated in Fig. 7d. Using Eq. (1), when IM = 8 mm and SM = 3 mm, the TM = 8.5 mm. Compared with the simple summation of TM = 11 mm, the TM = 8.5 mm was considered to be more reasonable for the positive side of the total error probability distribution. For the negative direction, when IM = 2 mm and SM = 3 mm, the TM = 3.6 mm. Compared with the simple summation of TM = 5 mm, our model was also reasonable. The TMs also seemed to be not too small from the probability distribution. This could be confirmed using another motion value. Therefore, our model of the margin determination was considered to be reasonable for both positional error compensation and suppressing the dose to surrounding normal tissues.

### Relationship between errors at positioning and margins

Setup errors of positioning by bony structures were almost 1 mm in 2*σ* in all directions. This shows that the accuracy of patient positioning in our system was high enough compared with the initial evaluation (Δ*P* of 2 mm in Table 1), and that the evaluated setup error can be reduced. However, errors of metal markers, which may be regarded as internal motion errors including positioning setup errors, arose. This could be because of (a) intra-fractional motion by fluctuation of the timings of gated-XTV shots and/or (b) inter-fractional motion errors. According to the histogram of metal marker errors, the errors were mostly distributed within 5 mm around 0 mm, with some large errors of more than 5 mm. The SDs for metal markers in Table 3 were influenced by such large errors. Least-square-fits of the marker error distribution to a normal distribution are shown in the bottom of Table 3, which represent the central spread of the distributions. The SDs were 1.4, 1.7, and 1.8 mm for the LR, SI, and AP directions, respectively. For the PTV setting, the values subtracting actual measured motion amounts from TMs, representing the whole margins excluding the expected actual motion within the gating, are shown in Table 4 as averages and SDs for all patients and directions, i.e., (2.5–3.0) ± (0.5–0.9) mm. The metal marker errors of the central spread were comparable to these margins. Although it is difficult to predict inter-fractional internal motion errors in advance, they do appear to be covered with our method for margins.

**Table 4:** Averages and SDs of the values subtracting actual measured motion amounts from TMs, representing the whole margins excluding the expected actual motion within gate for all patients

Direction	S	I	R	L	A	P
Average (mm)	3.0	2.6	2.8	2.7	2.6	2.5
SD (mm)	0.6	0.9	0.5	0.5	0.5	0.5

However, exceptionally large errors were not covered by these margins. The reason for this is considered to be changes in a patient’s physical conditions and/or tensions resulting in the observed large shifts of respiratory motion from the CT scans done for treatment planning. Repositioning the shifted target to the planned one is basically not permitted in particle beam radiotherapy because density changes, such as those due to bony structures in the beam path, may cause severe changes in the beam range and in the resulting dose distribution. Therefore, in such cases, confirmation of the dose distributions on the rescanned CT images using the planned beam settings is needed to help decide whether the treatment continues or re-planning is required. Also, further considerations of other techniques such as strict control of meal restrictions, improvement of patient fixation, and training of stabilized respiration before CT scan for treatment planning are required to suppress the changes in a patient’s physical conditions that cause poor reproducibility of respiratory motion. Acceptable amounts of shifts in patient positioning should also be considered for efficient daily operations.

### System delay

This paper proposes a method to derive appropriate timings of gated and 4D CT images including the delay times for gated radiotherapy. Without such delay times, an incorrect amount of motion during gated beam irradiation might be estimated using the mismatched images of different phases from the gated beam irradiation situation, leading to deriving larger or smaller margins than necessary. Comprehending these delay times is fundamentally important to obtain the XTV and CT images required to determine correct margins, appropriately, not only for ion beam therapy but for photon beam therapy. System delay times should be estimated at the commissioning stage to begin gated radiotherapy.

## Conclusion

We propose a strategy of individualized image acquisitions and treatment planning for respiratory-gated carbon-ion therapy, and we implemented it in clinical treatments for mobile organs such as the lungs at GHMC in June 2010. A treatment plan was made for each patient, which included margins in which gating motion was reflected on the basis of 4D CT images by taking into account the delay time of the gating system. From the treatment set-up images, distributions of positioning setup errors and internal motion errors of metal markers were obtained. The planned margins were considered to be mostly valid compared with the errors in many cases. Exceptionally large motion errors were observed in some cases, where further consideration should be given to continuing the treatment properly and efficiently.
